# In Situ Raman Study of Redox State Changes of Mitochondrial Cytochromes in a Perfused Rat Heart

**DOI:** 10.1371/journal.pone.0070488

**Published:** 2013-08-29

**Authors:** Nadezda A. Brazhe, Marek Treiman, Barbara Faricelli, Jakob H. Vestergaard, Olga Sosnovtseva

**Affiliations:** 1 Biophysics Department, Biological faculty, Moscow State University, Moscow, Russia; 2 Department of Biomedical Sciences, Faculty of Health and Medical Sciences, Copenhagen University, Copenhagen, Denmark; 3 The Danish National Foundation Research Center for Heart Arrhythmia, Copenhagen, Denmark; Scuola Superiore Sant'Anna, Italy

## Abstract

We developed a Raman spectroscopy-based approach for simultaneous study of redox changes in *c*-and *b*-type cytochromes and for a semiquantitative estimation of the amount of oxygenated myoglobin in a perfused rat heart. Excitation at 532 nm was used to obtain Raman scattering of the myocardial surface of the isolated heart at normal and hypoxic conditions. Raman spectra of the heart under normal pO_2_ demonstrate unique peaks attributable to reduced *c*-and b-type cytochromes and oxymyoglobin (oMb). The cytochrome peaks decreased in intensity upon FCCP treatment, as predicted from uncoupling mitochondrial respiration. Conversely, transient hypoxia causes the reversible increase in the intensity of peaks assigned to cytochromes *c* and *c1*, reflecting electron stacking proximal to cytochrome oxidase due to the lack of terminal electron acceptor O_2_. Intensities of peaks assigned to oxy- and deoxyhemoglobin were used for the semiquantitative estimation of oMb deoxygenation that was found to be of approximately 50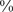 under hypoxia conditions.

## Introduction

Mitochondria are the main energy-producing organelles in eukaryotic cells and play a key role in regulation of cell survival and death [1], [2]. Redox reactions in mitochondria, in particular generation of reactive oxygen species (ROS), are essential to both of these aspects of mitochondrial function [1], [3]. Mitochondrial dysfunction is involved in a range of pathological conditions, including those representing the greatest disease burden of present time: cardiovascular diseases, neurodegeneration, diabetes and cancer [4]. In these diseases, an aberrant ROS production is typically encountered as a link between mitochondrial involvement and pathophysiology.

A great amount of research has been dedicated to understanding of the mechanisms and significance of ROS production by mitochondria, and there has been progress in methods allowing to monitor ROS production in living cells and whole organs [5], [6]. However, a thorough understanding of the mitochondrial function requires an ability to monitor — in an intact organ, and ultimately *in vivo* — the redox state of components of electron transport chain (ETC) determining the rates of ROS generation. This becomes particularly important in view of the fact that the redox state may be modified *in vivo* by a range of metabolic and hormonal influences [3], difficult to reproduce *in vitro*. Work on mitochondria isolated from organs subjected to pathological conditions or in cell-based disease models has uncovered functionally important changes in the redox state of ETC components. For instance, in mitochondria obtained from isolated, perfused rat hearts following acute ischemia, a loss of cytochrome *c* was observed [7], [8], with an increased reduction state of the portion of cytochrome *c* remaining in the mitochondria. Both of these alterations were shown to contribute to an increased ROS production in the course of ischemia [8]. In androgen-sensitive prostate cancer cells, reduced cytochrome *c* was found to be a substrate for p66Shc protein-mediated oxidation and ROS production underlying androgen-stimulated cell proliferation [9]. These examples point to the importance of studying redox state of ETC components in pathological conditions. To our knowledge, so far it has not been possible to carry out such studies in an intact organ, *in situ* or *in vivo*.

Raman spectroscopy is a promissing technique for studies of molecule vibrations in non-labelled live preparations. Non-elastic interaction of the incident light with molecule atoms results in the molecule transition between vibrational levels and Raman scattering of the light. Bond vibrations in a molecule and, therefore, the molecule Raman spectrum depends on the molecule conformation, its redox state, properties of the surrounding phase, etc. Hemoporphyrin-containing molecules (hemoglobin (Hb), myoglobin (Mb), cytochromes, etc.) possess intensive Raman scattering that is widely used for the assessment of the Fe ion spin and redox state and oxygenation [10]–[13]. Due to this hemoporphyrin spectral feature Raman spectroscopy provides unique possibility of label-free study of cytochrome redox state in mitochondria of living cells and tissues [14]–[16]. Raman scattering of cytochromes depends on the redox state of heme Fe atom [11], [17], [18]. Previously we demonstrated that by means of Raman spectroscopy it is possible to estimate semi-quantitatively the amount of reduced cytochromes *c, c1* and *b* in isolated live cardiomyocytes (CMs) and to distinguish functional state of ETC in mitochondria of healthy and pathological CMs [15]. We also observed a decrease in the amount of reduced cytochromes *c*, *c1* and *b* in live unlabeled CMs under hydrogen peroxide (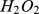) application [15].

In the present work, Raman spectroscopy was used to monitor the reduction state of mitochondrial cytochromes and the level of myoglobin oxygenation in a myocardium of isolated, perfused rat heart. We showed that Raman spectral peaks recorded from the subepicardial surface of heart muscle include peaks corresponding to mitochondrial cytochromes in isolated cardiomyocytes [15]. These peaks responded as expected to conditions known to affect the redox state of mitochondrial cytochromes: application of an uncoupler (carbonyl cyanide 4-(trifluoromethoxy)phenylhydrazone, FCCP), reducing agent (sodium dithionite, SDT) and hypoxia. Raman peaks attributable to oxy-or deoxymyoglobin (oMb and dMb, respectively) were identified and could be used to estimate myocardial Mb oxygenation. We propose that Raman spectroscopy may be applied to study, in an isolated heart, mechanisms of ETC damage. One important area of relevance for such studies is ischemic heart disease, a leading cause of death worldwide (approximately 7000000 cases per year [19]).

## Materials and Methods

### Preparation

Male Sprague Dawley rats, body weight 300–350 g (M&M Taconic, Denmark) were used. The animal studies conformed with the Guide for Care and Use of Laboratory Animals (National Institutes of Healt Publication No. 85-23, revised 1996) and Danish legislation governing animal experimentation, 1987, and were carried out after permission had been granted by the Animal Experiments Inspectorate, Ministry of Justice, Denmark.

#### Heart preapration

The rats were anesthetized with a mixture of hypnorm/midazolam/sterile water at the volume ratio 1: 1: 2 by s.c. injection (0.2 ml/100 g body weight). The animals were injected with heparin (1000 IE/Kg) through the femoral vein. Thoracotomy was performed and the heart was excised, and placed in a Petri dish containing ice-cold perfusion buffer. The heart was mounted on an aortic cannula attached to a perfusion system. The perfusion system consisted of a peristaltic pump (Biolab Ismatec) and a buffer reservoir connected to a water jacketed glass tubing with the aortic cannula attached at its outlet. Retrograde perfusion was started within 3 min of the heart excision with the flow rate set to 10–12 mL/min. Solutions were continuously gassed with 100% O_2_. Hearts were perfused with Tyrode solution containing (in mmol/l) 140 NaCl, 5.4 KCl, 5 HEPES, 1 Na_2_HPO_4_, 1 MgCl_2_, 1 CaCl_2_, 10 D-glucose (pH 7.4) at 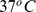. The perfusion system used here did not allow a recording of left ventricular pressure. However, the present procedure of heart removal and perfusion was the same as the procedure we have employed in several previous studies using Langendorff system, in which left ventricular developed pressure and heart rate were continuously recorded [20]–[22]. In these studies we have verified that a correct attachment of the heart to the perfusion cannula invariably resulted in a resumption of a regular contractile activity, as confirmed visually in the present work. The heart was resuming regular contractions upon perfusion with Ca^2+^ containing oxygenated warm Tyrode buffer. After 10–15 min of heart perfusion with this solution the contractions were arrested by switching the perfusion to Ca^2+^-free Tyrode solution in order to enable recording of Raman spectra.

#### Cardiomyocyte preparation

Isolated cardiomyocytes were prepared by enzymatic dissociation during retrograde perfusion of the heart using a modified Langendorff technique as described in [15], [23]. Briefly, rats were anesthetized with a mixture of hypnorm/midazolam/sterile water (1∶1∶2) by s.c. injection (0.25 ml/100 g body weight) and heparinized (5000 U/ml i.m., 0.7–1 ml/rat) for 10 min. Rat hearts were excised, mounted on a Langendorff system, and perfused through the aorta for 35 min using oxygenated, Ca^2+^-supplemented Tyrode solution containing (in mmol/l) 140 NaCl, 5.4 KCl, 10 HEPES, 1 MgCl_2_, 1 CaCl_2_, and 10 D-glucose (pH 7.4) at 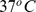, then with Ca^2+^-free Tyrode solution for 7 min before digestion with 50 ml of the same solution containing collagenase (type 4, 50 mg, 300 units/mg) and protease (type XIV, 0.2 mg/ml), recirculated for 16–18 min. Collagenase was subsequently removed by perfusion with Ca^2+^-free Tyrode solution at pH 7.4 for 7 min. All solutions were gassed with 100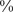 O_2_ for 5 min before use. The atria and blood vessels were then removed, and the free ventricles were minced into small pieces. Cells were dissociated by gentle mechanical shaking. Isolated CM were then incubated in Ca^2+^-free Tyrode solution with 1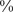 BSA and 20 mmol/L 2,3-butanedione monoxime (BDM) at room temperature for 20 min. The concentration of Ca^2+^ was gradually increased up to 1 mmol/l during the next 25 min. Cell viability and concentration were determined by Trypan blue assay. Typically, we obtained a 60–80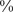 yield of quiescent, rod-shaped CM with the total yield from one heart 90–100×10^5^ cells. Pyruvate 2 mM was added to the CM suspension before measurements.

#### Mitochondria preparation

Mitochondria were prepared using a Polytron method of Pasdois et al. [8] with minor modifications. Briefly, each heart was homogenized on ice in 6 ml of a buffer consisting of (in mmol/l) 10 Tris-HCl, 2 EGTA, 300 sucrose, 0.5 phenyl-methyl-sulphonylfluoride, aprotinin 10 µg/ml (pH 7.1), using a sequence of two rotating blade homogenizers (10 strokes with Ultra Turrax, Bie
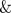
Berntsen, DK; followed by 10 seconds with Polytron PT1200, Holm
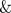
Halby, DK). The homogenate was diluted with equal volume of the same buffer and centrifuged at 700 g in 10 min, followed by a centrifugation of the supernatant at 7000 g in 10 min. The pellets, representing a mitochondria-rich fraction, were washed at 7000 g in 10 min in a combined volume of 8 ml, resuspended in a final volume of 2 ml of the same buffer and stored at 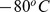.

### Raman spectroscopy study

**Figure 1:**
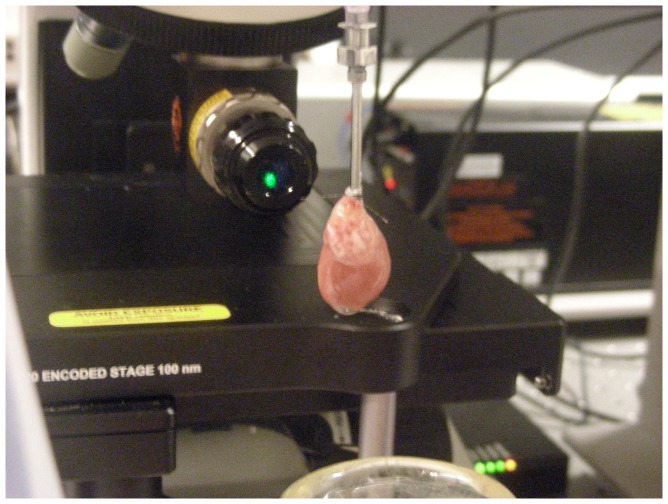
Heart position with respect to the objective and laser light. An isolated heart is attached to the perfusion system through an aortic cannula for retrograde perfusion. Excitation and registration of the Raman scattering is done through the objective.

Raman spectra were recorded from the epicardial surface of the left ventricle with InVia Raman microscope (Renishaw, UK) with 532 nm laser using macrokit attachment(Renishaw, UK) with the focusing lens of 60 mm focusing distance. Laser power was 3 mW per registration spot with 40 µm diameter. Raman spectra of heart in all experiments were collected for 5 min. The position of the heart with respect to the objective is shown in Fig. 1.

Raman spectra of live CMs and isolated mitochondria were collected for 20 s using 63× water immersion objective (Leica) with NA of 0.9 [15]. Laser power was 0.15 mW per registration spot with 400 nm diameter. Raman spectra of purified Mb and cytochrome *c* were recorded using macrokit attachment with a lens of 60 mm focusing distance and the laser power 0.3 mW per registration spot with 40 µm diameter. Spectra were collected for 20 s.

Raman spectra were processed using open source software [24]. Baseline was subtracted in each spectrum. The parameters for baseline subtraction were chosen after the processing of approximately 50 spectra from different hearts to ensure that all baseline variations were taken into account.

#### FCCP and SDT application

To study dependence of Raman peaks on the mitochondrial membrane potential, the protonophore FCCP was added into the perfusing solution in a final concentration of 10^−5^ M. To reduce all mitochondrial cytochromes and to produce oMb deoxygenation we added a small amount of sodium dithionite into the perfusing solution. All chemicals were purchased from Sigma.

#### Global stop-flow ischemia

Global ischemia was induced by stopping the perfusion for 35 min. Spectra were collected twice before ischemia, at 15 and 30 min of ischemia and then at 5 and 30 min of reperfusion. In experiments to check the effect of the long-term Raman measurements on the heart Raman spectra were recorded at 0, 5, 25, 40, 50 and 75 min after the beginning of the experiment.

Both global stop-flow hypoxia and SDT application result in the decrease of tissue pO_2_ that is more severe in SDT experiments, than in stop-flow hypoxia experiments since SDT reacts with O_2_ and eliminates it from the perfusion solution and the heart tissue. Besides, SDT acts as a direct reducer of cytochromes causing complete reduction of the whole cytochrome pool in mitochondria. SDT also directly interacts with oMb causing its deoxygenation. Thus, in global stop-flow ischemia experiments any change in the amount of reduced cytochromes and oMb is expected to be mediated by lack of tissue O_2_, whereas in SDT experiments these changes are expected to be caused mainly by a direct interaction of SDT with oxidized cytochromes and oxymyoglobin.

## Results and Discussion

### Peak assignment in Raman spectrum of the whole heart

To perform assignment of peaks in Raman spectrum of the whole heart we compare Raman spectra of the heart, isolated cardiomyocytes and mitochondria with and without SDT application and Raman spectra of putified reduced and oxidized cytochrome *c*, oxy-deoxy-and metmyoglobin.

**Figure 2:**
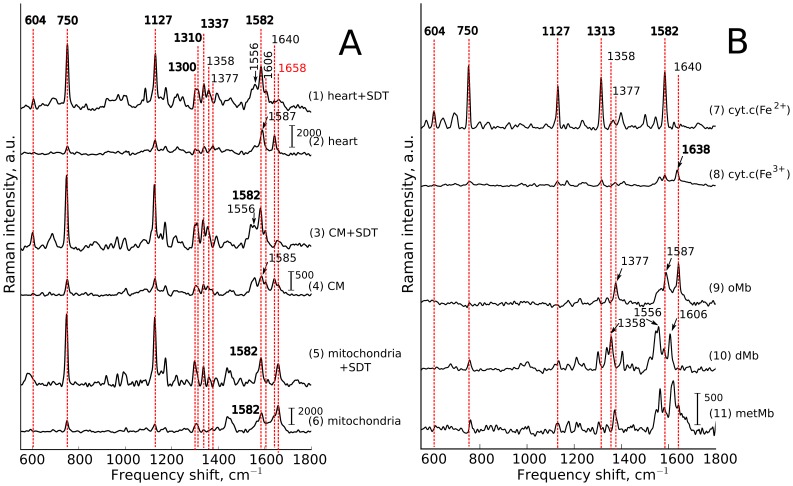
Peak assignment. (A): Assignment of peaks in Raman spectra of perfused heart, cardiomyocytes (CMs) and isolated CM mitochondria in partially oxidized state and after reduction with sodium dithionite (SDT). (B): Assignment of peaks in Raman spectra of reduced (cyt.c(Fe^2+^)) and oxidized (cyt.c(Fe^3+^)) cytochrome *c*, oxymyoglobin (oMb), deoxymyoglobin (dMb) and metmyoglobin (metMb). Vertical scale bars show Raman intensity, a.u. In Fig. 2.A vertical scale bars are the same (2000 a.u.) for heart and mitochondria with and without SDT and the scale bar is equal to 500 a.u. for cardiomyocytes with and without SDT. In Fig. 2.B scale bar (2000 a.u.) is the same for all spectra. Numbers above red dashed lines indicate peak positions, cm^−1^. Arrows with numbers show position of peaks that are shifted relatively to the dashed line. Numbers indicating positions of peaks corresponding to cytochromes *c*, *c1* and *b* are shown in bold font, to myoglobin (oMb and dMb) are shown in regular font. Peak with position at 1658 cm^−1^ (shown in red-colored font) corresponds to 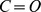 bond vibration in peptide backbones of protein 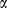-helixes.

Laser excitation at 532 nm evokes resonance Raman scattering of heme-containing molecules such as cytochromes and myoglobin (Mb). Since the heme structure in cytochromes and Mb is almost the same their Raman spectra possess sets of peaks with similar positions of frequency shifts. However, since the surrounding protein in these molecules differs and since myoglobin is cytosolic molecule and cytochromes are transmembrane (cytochromes *b* and *c1*) or membrane-bound proteins (cytochrome *c*), the relative input of peak intensities into overall Raman spectrum is also different [12], [15]–[17]. To identify the main peaks in spectra from the heart, we also recorded Raman spectra of isolated CMs and isolated heart mitochondria (Fig. 2A), as well as of purified oxidized and reduced cytochrome *c*, oxy-, deoxy-, and methemoglobin (oMb, dMb and metMb, respectively) (Fig. 2B). Under 532 nm excitation conditions, the contribution of the oxidized cytochromes *b* and *c* to Raman scatter is negligible [17]. Therefore, to aid cytochromal peak identification we used sodium dithionite in some experiments to achieve maximal cytochrome reduction. Likewise, SDT aids in an assignment of Mb peaks, due to a frequency shift upon transition from oMb to dMb.

Raman spectra of the heart have main peaks with maximum positions at 750, 1127, 1587, 1640 cm^−1^ under partially oxidized conditions, and at 750, 1127, 1582 cm^−1^ after SDT application. There are also less intensive peaks located at 1300, 1310, 1337, 1377 cm^−1^ under partially oxidized conditions, and 604, 1300, 1310, 1358, 1556 and 1606 cm^−1^ after SDT application. It can be seen that this set of peaks is almost the same as the set of peaks in Raman spectra of CMs, heart mitochondria, isolated cytochromes and myoglobin (Fig. 2, Table 1). There are some other peaks in the region of 900–1300 cm^−1^, however we shall not consider them in this paper.

**Table 1:** Comparison of peaks in Raman spectra of the whole heart, reduced or oxydized purified cytochrome c and purified oxy-or deoxymyoglobin.

Heart			Cytochrome c		Myoglobin		Comments
Control*	+SDT*	Contribution from**	Cyt.*c*(*Fe* ^3+^)	Cyt. *c*(*Fe* ^2+^)	dMb	oMb	
—	604	cyts.c,c1(Fe^2+^)	—	604	—	—	Unique peak of cyts.c,c1(Fe^2+^)
750	750 ↑	cyts.c,c1(Fe^2+^), cyts.b(Fe^2+^)	750	750 ↑	750	—	SDT-induced increase of the peak intensity is a signature of cyts.
1127	1127 ↑	cyts.b(Fe^2+^), cyts.c (Fe^2+^)	1127	1127 ↑	—		
1300–1310	1300–1310 ↑	**cyts.c,c1,b(Fe^2+^)**	1313	1313	—	—	In cyts.b the peak maximum locates at 1300 cm^−1^ [25]
1337	1337 ↑	**cyts.b(Fe^2+^)**	—	—	—	—	Unique peak of cyts.b(Fe^2+^)
—	1358(a)	dMb	—	—	1358(a)	—	Ratio of peak intensities at 1358 and 1377 cm^−1^ can be used to estimate relative dMb amount.
1377(a)	—	oMb	—	—	—	1377(a)	
	1556(b)	**dMb**, oMb	—	—	1556(b) ↑	1556(b)	In heart under control conditions the spectrum range 1550–1640 cm^−1^ originates from oMb, whereas under SDT-reduction — from reduced cytochromes (1582 cm^−1^) and dMb (1556 and 1606 cm^−1^)
—	1582	cyts.c,c1,b(Fe^2+^)	1582	1582 ↑	—	—	
1587(b)	—	**oMb**, dMb	—	—	1587(b)	1587(b) ↑	
—	1606(c)	dMb	—	—	1606(c)	—	
1640(c)	—	oMb	1638	—	—	1640(c)	

* The same set of peaks we observe in Raman spectra of cardiomyocytes and isolated CM mitochondria;

** Based on [11], [16], [17], [25] and our own observations;

(a) — symmetric pyrrol half-ring vibrations of Mb heme (A1g/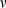
4 symmetry), sensitive to the Redox state of heme Fe and presence of O_2_;

(b) and^(c)^— vibrations of heme methine-bridges (A1g and B1g/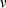
10 symmetry, respectively), sensitive to the spin state of heme Fe and diameter of the heme ring.

Numbers indicate positions of peak maxima (cm^−1^). Arrows mark peaks whose intensity significantly increases in Raman spectra of the heart after SDT application, reduction of cytochrome c, or under binding or release of O_2_ from myoglobin. Cytochrome or Mb type indicated in bold font is the main contributer to the Raman scattering of the heart at the designated frequency shift.

#### Cytochromal peaks

Addition of SDT to the perfusion solution causes the reduction of all cytochromes leading to the increase in the intensity of their Raman scattering. Thus, significant increase in the intensity of peaks at 750, 1127, 1300, 1310 and 1337 cm^−1^ in the Raman spectrum of the heart is observed (Fig. 2.A, traces 1, 2, Table 1). In addition, a peak at 604 cm^−1^ appears. The same changes occur in Raman spectra of CMs and heart mitochondria after SDT application (Fig. 2.A, traces 3–6) evidencing the cytochromal origin of the listed peaks in the heart spectra. Since 532 nm laser excitation causes Raman scattering from *c* and *b*-type cytochromes, in Raman spectra of the whole heart, CMs and mitochondria, cytochromal peaks originate from both cytochrome types. Comparison of Raman spectra of the whole heart and purified cytochrome *c* (Fig. 2, traces 1, 2 and 7, 8, Table 1) and literature data [25] show that in Raman spectra of the heart there are cytochromal peaks common to *c*, *c1* and *b* cytochromes and specific peaks corresponding to cytochromes *c*, *c1* or cytochromes *b*. Thus, the major peaks at 750 and 1127 cm^−1^ are present in both types of cytochromes, whereas the peaks at 604 and 1310 cm^−1^ correspond to *c*-type cytochromes and the peaks at 1300 and 1337 cm^−1^ — to *b*-type cytochromes. We should note that peak at 1313 cm^−1^ observed in pure cytochrome *c* spectra is downshifted to 1310 cm^−1^ in heart spectra. This can be due to its overlapping with cytochrome *b* the peak at 1300 cm^−1^ that causes slight shift of 1313 cm^−1^ peak maximum position.

Raman spectra of SDT-treated heart and SDT-treated CMs, both SDT-treated and SDT-nontreated mitochondria, reduced and oxidized purified cytochrome *c* demonstrate an intensive peak at 1582 cm^−1^ (Fig. 2, traces 1, 3, 5–8). Note that in SDT-treated heart, SDT-treated CMs and reduced cytochrome *c* its intensity is approximately the same as intensity of the peaks at 750 and 1127 cm^−1^ (Fig. 2, traces 1, 3, 7). However, in hearts not treated with SDT the peaks at 1587 and 1640 cm^−1^ appear. This effect is due to the fact that in normal SDT-nontreated heart the peaks at 1587 and 1640 cm^−1^ originate from oMb. Cytochromal peak at 1582 cm^−1^ is much less intensive in the oxidized cytochromes and therefore it does not affect intensity and position of oMb 1587 cm^−1^ peak. After SDT treatment of the heart and CMs cytochromal peak intensities increase and oMb transits to dMb. As a result, oMb peaks at 1587 and 1640 cm^−1^ shift to dMb peaks at 1556 and 1606 cm^−1^, respectively (Fig. 2, traces 1, 3, 10, table 1) and cytochromal peak at 1582 cm^−1^ can be clearly seen (Fig. 2, traces 1 and 3). Since there is no Mb in isolated heart mitochondria a peak at 1582 cm^−1^ is observed in both SDT-treated and partially oxidized preparations. Remarkably, in Raman spectra of isolated mitochondria we can see a peak at 1658 cm^−1^ corresponding to the vibration of *C = O* bond in the peptide backbones of protein 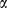-helixes [26] (Fig. 2, traces 5 and 6).

Importantly, peaks at 750 and 1127 cm^−1^ in Raman spectra of the heart, CMs and mitochondria represent both *c* and *b*-types of cytochromes. However, relative contributions of these peaks to the overall Raman spectrum differ for cytochromes *c*, *c1* and cytochromes *b*. Thus, Adar et al. [11] showed that under 530.9 nm laser excitation the Raman peak at 750 cm^−1^ was mainly determined by *c*-type cytochromes, whereas peak at 1127 cm^−1^ by *b*-type cytochromes. Hence, the ratio of intensities 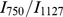 can be used to estimate the relative amount of reduced cytochromes *c*, *c1 vs.* reduced cytochromes *b*.

#### Myoglobin peaks

As the experiments were carried out under conditions with arrested heart contraction, most of Mb molecules (no less than 90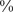) were expected to be in oxygenated state (oMb) [27]. Indeed, Raman spectrum of the heart under normal oxygen tension demonstrates a set of peaks corresponding to heme vibrations in oMb: 1377, 1587 and 1640 cm^−1^ (Fig. 2, traces 2 and 9, Table 1). The same peaks can be seen in the spectra of CMs (Fig. 2, trace 4). Application of SDT causes deoxygenation of the perfusion solution and evokes oMb transition to dMb. This leads downshift of oMb peaks to 1358, 1556 and 1606 cm^−1^, respectively, (Fig. 2, traces 1, 3 and 10, Table 1). The peak at 1638 cm^−1^ presents in Raman spectrum of oxidized cytochrome c (Fig. 2, trace 8). However, considering the fact that only reduced cytochromes contribute to the heart and CM Raman spectra, the 1640 cm^−1^ peak visible in the heart and CM spectra under normal O_2_ tension conditions may be considered as oMb peak.

### Reduction state of mitochondrial cytochromes during protonmotive force collapse and ischemia

In respiring mitochondria, the reduction state of ETC redox centers is highly dynamic and depends on a number of changing conditions, including oxygen and substrate supply, metabolic demand and hormonal status. Because of the dependence of proton pumping across the inner mitochondrial membrane on the electron transport, the latter being governed ultimately by the redox potential differences along the ETC, changes in the proton gradient (the protonmotive force) exert a strong effect on the status of the ETC redox centers [1]. To test whether the main Raman peaks identified above behave in accordance with their mitochondrial or myoglobin origin, we measured myocardial Raman spectra following dissipation of the protonmotive force by FCCP, or following an oxygen deprivation by a stop-flow ischemia.

#### The effect of dissipating the mitochondrial protonmotive force

**Figure 3:**
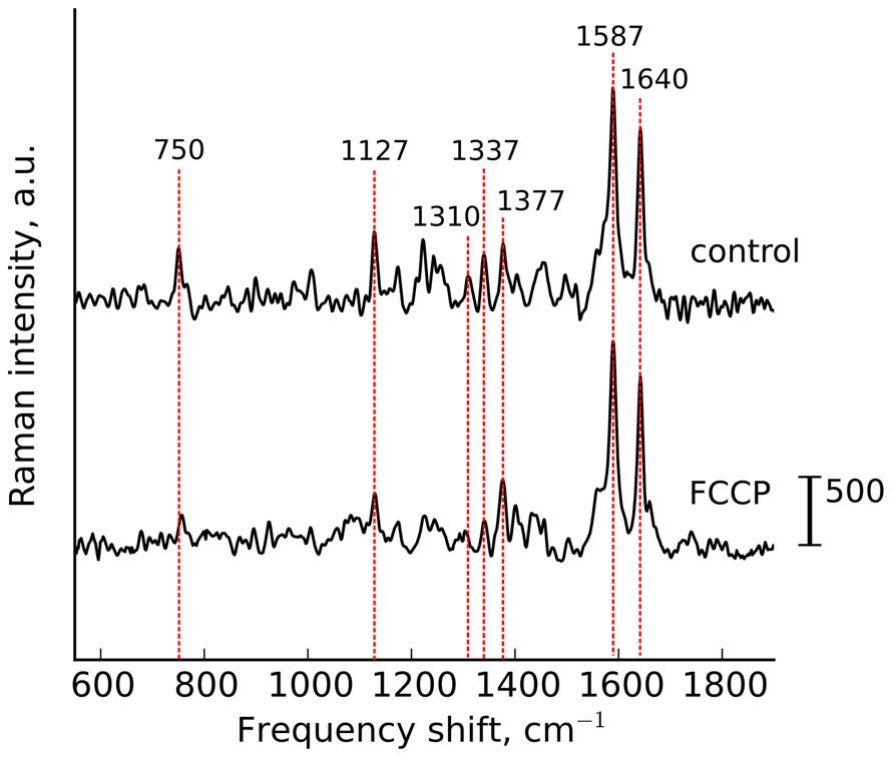
FCCP application. Raman spectra of perfused heart in control and under the application of protonophore FCCP (10 *μ*M).

If the protonmotive force is dissipated by an increased proton re-entry to the mitochondrial matrix (such as in ADP-stimulated respiration or uncoupling), the electron flow along the ETC increases with a concomitant decrease of the reduction of electron carriers including cytochromes [28], [29]. Accordingly, we expected an addition of FCCP to the perfusion solution to cause a decrease in the intensity of cytochromal peaks. Fig. 3 shows that peaks at 750, 1127, 1310 and 1337 cm^−1^ all diminished in amplitude following FCCP application. In contrast, there were no changes in the intensities of peaks at 1377, 1587 and 1640 cm^−1^ because of constant amount of oMb. Peak heights at 750 and 1127 cm^−1^, expressed relative to the sum of all intensities within the spectra (Table 2) were significantly decreased at 5 and 10 min of FCCP treatment. We conclude that the behavior of the designated mitochondrial cytochromal Raman peaks following FCCP treatment was in agreement with the expected dependence of the reduction state of *c*-and *b*-type cytochromes on the protonmotive force.

**Table 2:** Relative intensity of cytochromal peaks normalized to the sum of the whole spectrum intensities in normal perfused heart and under application of FCCP.

	*I*_750_/*I_sum_*	*I*_1127_/*I_sum_*	*I*_750_/*I*_1127_
Control heart	100±9	100±2.3	103.8±2.43
Heart+FCCP, 5 min	56.7±2.7^*^	42.3±3.6^*^	126±15
Heart+FCCP, 10 min	61.5±12.7^*^	37.3±2.3^*^	150±43

Data are shown as mean values
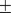
SE (n = 3). Nonparametric Kruskal-Wallis test with post Dunns multiple comparison test gives 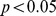 between data of control experiments and experiments with FCCP application (^*^).

#### The effect of oxygen deprivation

**Figure 4:**
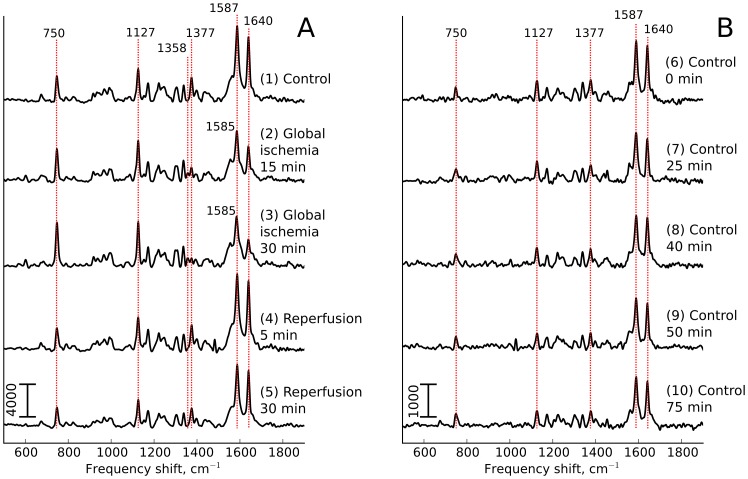
Experiment on global ischemia. (A): Raman spectra of the isolated heart under global stop-flow ischemia. (B) Raman spectra of the control heart continuously perfused during the time interval equaled to the duration of global ischemia experiment. Registration time points in control experiment correspond to the registration time points in global ischemia experiment.

**Figure 5:**
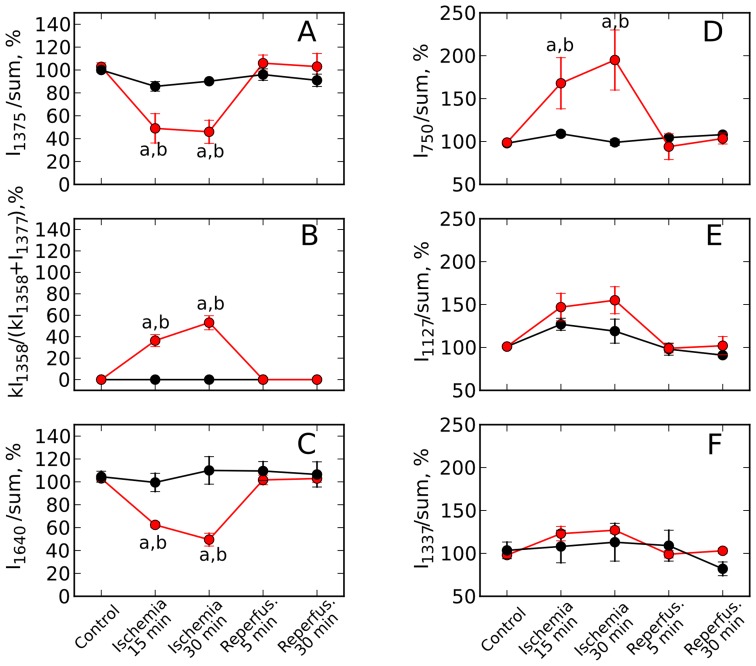
Effect of global ischemia on the amount of reduced cytochromes *c, c1, b* and oMb. Effect of global stop-flow ischemia on the normalized intensity of oMb peaks at 1375 and 1640 cm^−1^ (A and C), on the relative amount of dMb (B) and on the normalized intensities of peaks corresponding to reduced cytochromes *c, c1* and *b* at 750 and 1127 cm^−1^ (D and E), and to cytochromes *b* at 1337 cm^−1^ (F). Red line and markers correspond to the data of global ischemia experiments, black line and markers — to the data of control experiments. Normalization of peaks was done to the sum of the whole spectrum intensities. Values are mean
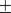
SE (n = 3). Nonparametric Kruskal-Wallis test with post Dunns multiple comparison test gives 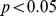 between results for 0 min and ischemia (15 and 30 min) (*a*) and for 15 and 30 min of ischemia and corresponding time in the control experiment (*b*).

Global ischemia was applied by stopping the perfusion for 35 min, and Raman spectra were recorded starting at 15 and 30 min. These hypoxic conditions caused changes in myoglobin peak intensities (Fig. 4, left side, traces 2 and 3): oMb peaks at 1377 and 1640 cm^−1^ decreased progressively at 15 and 30 min ischemia, with an appearance of the dMb peak at 1358 cm^−1^ (Fig. 4, left side, traces 2 and 3). We should note that dMb Raman scattering at the 532 nm excitation is approximately 50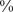 less intensive than that of oMb resulting in the relatively low intensity of dMb peak at 1358 cm^−1^. These changes were consistent with gradually diminishing cardiomyocyte pO_2_ during the stop-flow period. At the same time there were progressive increase in the intensity of 750 cm^−1^ peak and the appearance of 604 cm^−1^ peak associated predominantly (750 cm^−1^) and exclusively (604 cm^−1^) with cytochromes *c1* and *c* (Fig. 4, left side, traces 2 and 3). The minor 604 cm^−1^ peak was previously distinguished only in the presence of SDT (Fig. 2, trace 1)). In contrast, peaks associated predominantly (1127 cm^−1^) or exclusively (1337 cm^−1^) with cytochromes *b* appeared to show only a small or no increase (Fig. 4, left side, traces 2 and 3). To quantify these changes, we expressed peak heights relative to the sum of all intensities within the measured spectra. This normalization revealed a significant, time-dependent decrease in the amount of oMb during ischemia period, returning to the preischemic level upon reperfusion (Fig. 5.A, C). Figs. 5. D–F show time-dependent changes of similarly normalized values for cytochromal peaks. At 30 min ischemia time, there was a statistically significant doubling of the relative intensity of 750 cm^−1^ peak (mainly representing cytochromes *c1* and *c*), with no significant change of the relative intensities of peaks at 1127 and 1337 cm^−1^ (representing b cytochromes). As observed for the Mb peaks, the relative intensities of the cytochromal peaks returned to the preischemic levels within 5 min of reperfusion.

It is known that in oxygen-binding proteins like Mb and hemoglobin (Hb) 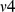 peak (peaks with position at 1377/1358 cm^−1^ in oMb/dMb and 1375–1377/1355–1357 cm^−11^ in oHb/dHb) is sensitive to the redox state of heme Fe and presence of O_2_ [12]. Ward et al. [13] demonstrated that the ratio of the peak intensities 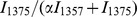 could be used for the monitoring of blood oxygenation in tissue vasculature. 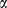 was an empirically derived parameter accommodating the difference in Raman scattering intensities of oHb and dHb. Parameter 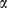 was calculated as the ratio of intensities 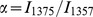, where 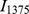 and 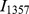 were estimated as maximal intensities of 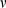
4 peak in solutions of purified oHb or dHb, respectively.

Analogous to this approach we propose that ratio 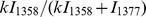 can be used for a quantitative estimation of the relative amount of dMb in the whole heart (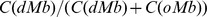). Here values 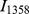 and 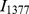 are estimated as the maximal intensity of corresponding peaks in the Raman spectrum of the heart under conditions of interest and the parameter 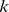 represents the difference in the Raman scattering intensity of 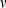
4 peak in oMb and dMb. For this purpose the parameter 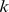 was calculated as the ratio 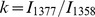, where 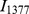 and 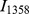 were maximal intensities of 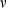
4 peak in Raman spectra of hearts with fully oxygenized Mb and of hearts treated with SDT. Parameter 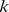 was calculated as the mean value from three independent experiments and was equaled to 1.55
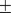
0.1 (mean
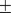
SE).

Fig. 5B showes that the ratio 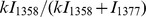 reached approximately 50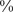 at 30 min ischemia. This would suggest a cardiomyocyte pO_2_ at this time point to be near P_50_ value for Mb, or approximately 3 mmHg [27], [30] This value is consistent with our experiments conducted on the arrested hearts, with O_2_ consumption expected to be markedly smaller than in contracting hearts. In a beating rat heart, 10 min of global ischemia was shown to cause a pO_2_ decrease to less than 1 mm Hg [31].

Hypoxia caused an increase in the fractional reduction of respiratory complexes upstream from cytochrome oxidase [32], [33]. In rat liver mitochondria, Wilson et al. [33] have shown an increasing degree of cytochrome *c* reduction when decreasing O_2_ concentrations to below 30 µM (approximately 20 mm Hg). Likewise, cytochrome *c* reduction was increased in mitochondria isolated from rat hearts at the end of 30 min of global ischemia [8]. We propose that the increase in the relative intensity of 750 cm^−1^ peak (representing mostly cytochromes *c1* and *c*, Figs. 4 and 5) reflects the same phenomenon. Although measurements of redox environment *in vivo* have been performed using Electron Paramagnetic Resonance [6], to our knowledge the present application of Raman spectroscopy has assessed for the first time changes in the reduction state of specific cytochromes in an intact organ. In contracting hearts, ischemia of 20–30 min duration damages several of the respiratory complexes, including complex III [34], [35]. This ischemia-induced damage to complex III involves the Rieske iron-sulfur protein (ISP) of the complex. The impairment of the electron transfer through the ISP leads to an increased reduction of cytochrome *b* [7], [34], in analogy to a blockade of complex III by antimycin A. Since there is no corresponding increase in the reduction state downstream at cytochromes *c1* and *c*, an ischemia-induced damage to complex III would lead to an increased ratio of reduced cytochromes *b*/reduced cytochromes *c*. In contrast, the data in Fig. 5. D–F indicate a decrease in this ratio. We therefore conclude that under present conditions (30 min global ischemia in arrested hearts), no measurable damage to complex III has occurred. This conclusion is consistent with the cytochromal redox changes reverting fully to the preischemic values upon reperfusion (Fig. 4, traces 4 and 5 and Fig. 5, D–F).

## Conclusion

We used Raman spectroscopy to monitor changes in the redox state of the mitochondrial cytochromes and level of Mb oxygentation in an isolated, perfused rat heart. We identified the major Raman peaks as contributed mainly by reduced cytochromes *c* and *c1* (at 750 cm^−1^) and reduced cytochromes *b* (at 1127 cm^−1^). A number of less intensive peaks were also assigned to specific cytochromes (Table 1), including peaks unique for cytochromes *c*, *c1* (604 cm^−1^) and *b* (1337 cm^−1^). Major peaks assigned to oxymyoglobin were present in the obtained Raman spectra: peaks at 1587 and 1640 cm^−1^ that were shifted to 1556 and 1606 cm^−1^, respectively, under oMb deoxygentation and transition to dMb. Myoglobin peaks were shown to respond to the changes in ambient pO_2_. In analogy with previous studies on hemoglobin [13], we propose to estimate the relative Mb deoxygentation on the basis of intensities of those Raman peaks uniquely representing oMb (1377 cm^−1^) and dMb (1358 cm^−1^). The advantage of the proposed Raman-based approach is that the registration of Raman scattering from oMb and dMb in the whole heart and the semi-quantitative estimation of the relative oMb amount allows to estimate pO_2_ in the heart tissue.

By means of Raman spectroscopy we observed that under normoxic conditions in a an isolated, contracted-arrested heart the mitochondrial cytochromes were in partially reduced state and responded dynamically to changing conditions: amount of reduced cytochromes decreased following the uncoupling of respiration (Fig. 3), and increased (cytochromes *c1* and *c*) under hypoxia (Fig. 4). In hypoxia, the increase (reversible with reperfusion) in the ratio of the amount of reduced cytochromes *c1*, *c* over cytochromes *b* (Fig. 5) suggested that under our conditions no ETC damage occurred, as discussed above.

This study has some limitations. First, the main limitation is a necessity to maintain a stable focus distance that results in the use of an arrested heart. This lack of contractile activity during Raman spectrum recordings would decrease the rate of ATP and O_2_ consumption, and is expected to prolong the hypoxia period after which the mitochondrial redox changes would still be reversible. The present results suggest that longer experimental ischemia time will be necessary in the future, if a complete tissue anoxia is to be achieved. Secondly, Raman spectra were collected from the subepicardial surface of left ventricle, from an area with diameter of approximately 40 µm. While there was a good qualitative agreement between these spectra and those of isolated cardiomyocytes, suggesting the subepicardial recordings to be representative of the entire myocardium, some quantitative heterogeneity might conceivably be revealed by multiple sampling points, including points at the endocardial surface. We would like to note that in the present study these limitations were not crucial since our goal was to demonstrate possibility of monitoring of redox state changes occurring in mitochondrial *b*-and *c*-type cytochromes and the possibility of estimation of Mb oxygenation in the living heart under hypoxia. Besides, listed limitations can be overcome in future by usage of the specialy designed optic fiber equiped with an objective with high numerical aperture.

In conclusion, we would like to emphasise out that the intact heart measurements provide valuable information that can be lost in experiments with isolated cardiomyocytes. Studies in recent years have revealed some new properties of the intact heart as a system more complex than the individual cells, and one endowed with some unique characteristics [36], [37]. Findings of Davidson and colleagues [37] suggest strongly that mitochondrial redox state is under influence of the myocardial syncytium, and that measurements of electron transport chain redox state should be made in an intact system to obtain a more realistic reflection of mitochondrial function as part of the entire network. Present study of isolated perfused heart by means of Raman spectroscopy illustrates potential of this technology in studies of redox changes of mitochondrial ETC components in the intact organ. In the specific context of heart ischemia, Raman spectroscopy seems to offer new insights into ETC redox state *in situ*, and to allow more precise monitoring of hypoxia effects, as well as of therapeutic interventions.
